# Volatile Compound Screening Using HS-SPME-GC/MS on *Saccharomyces eubayanus* Strains under Low-Temperature Pilsner Wort Fermentation

**DOI:** 10.3390/microorganisms8050755

**Published:** 2020-05-18

**Authors:** Kamila Urbina, Pablo Villarreal, Roberto F. Nespolo, Ricardo Salazar, Rocio Santander, Francisco A. Cubillos

**Affiliations:** 1 Departamento de Biología, Facultad de Química y Biología, Universidad de Santiago de Chile, 9160000 Santiago, Chile; kamila.urbina@usach.cl (K.U.); pablo.villarreal.d@usach.cl (P.V.); 2 Millennium Institute for Integrative Biology (iBio), 7500565 Santiago, Chile; robertonespolorossi@gmail.com; 3 Instituto de Ciencias Ambientales y Evolutivas, Universidad Austral de Chile, 5090000 Valdivia, Chile; 4 Center of Applied Ecology and Sustainability (CAPES), 8331150 Santiago, Chile; 5 Laboratorio de Electroquímica del Medio Ambiente, LEQMA, Departamento de Química de los Materiales, Facultad de Química y Biología, Universidad de Santiago de Chile, 9160000 Santiago, Chile; ricardo.salazar@usach.cl; 6 Laboratorio de Cinética y Fotoquímica, Departamento de Ciencias del Ambiente, Facultad de Química y Biología, Universidad de Santiago de Chile, 9160000 Santiago, Chile; rocio.santanderm@usach.cl

**Keywords:** yeast, volatile compounds, *S. eubayanus*, beer, lager

## Abstract

The recent isolation of the yeast *Saccharomyces eubayanus* has opened new avenues in the brewing industry. Recent studies characterized the production of volatile compounds in a handful set of isolates, utilizing a limited set of internal standards, representing insufficient evidence into the ability of the species to produce new and diverse aromas in beer. Using Headspace solid-phase microextraction followed by gas chromatography-mass spectrometry (HS-SPME-GC-MS), we characterized for the first time the production of volatile compounds in 10 wild strains under fermentative brewing conditions and compared them to a commercial lager yeast. *S. eubayanus* produces a higher number of volatile compounds compared to lager yeast, including acetate and ethyl esters, together with higher alcohols and phenols. Many of the compounds identified in *S. eubayanus* are related to fruit and floral flavors, which were absent in the commercial lager yeast ferment. Interestingly, we found a significant strain × temperature interaction, in terms of the profiles of volatile compounds, where some strains produced significantly greater levels of esters and higher alcohols. In contrast, other isolates preferentially yielded phenols, depending on the fermentation temperature. This work demonstrates the profound fermentation product differences between different *S. eubayanus* strains, highlighting the enormous potential of this yeast to produce new styles of lager beers.

## 1. Introduction

Beer is the most popular and widespread alcoholic drink in the world, offering a wide variety of styles and flavors around the globe [1]. The production of this beverage is determined by well-defined processes, such as malting, grinding and macerating the grains, cooking the wort, and finally fermenting [2]. The final fermented product contains unique organoleptic properties, as a result of a delicate balance between aromas and flavors provided by the main ingredients used during the process: malt, water, hops, and yeast [3,4].

Industrial beer production is dominated by yeasts belonging to the *Saccharomyces* genus, mainly *Saccharomyces cerevisiae* responsible for ale beer fermentation, while *S. pastorianus* (a hybrid between *S. cerevisiae × S. eubayanus*) is used for lager beers. Unlike ales, which are fermented by *S. cerevisiae* in a range of 16–25 °C, lagers are fermented by *S. pastorianus* at lower temperatures (8–15 °C), mostly because of the cryotolerant contribution of *S. eubayanus* to the lager hybrid [5]. Lager beer represents more than 90% of the beer produced worldwide and is the most popular style consumed [6]. During fermentation, yeast cells produce a wide range of secondary metabolites, including volatile compounds (VCs) responsible for the complex flavors and aromas found in beer. VCs are molecules with low molecular weight and rapid dispersion in the environment and determine the different organoleptic qualities of beer [7,8]. Some of the VCs found in beer are synthesized by yeast cells through several metabolic pathways, emphasizing that a significant contribution to the organoleptic properties of beer is provided by the particular strain used for fermentation [2]. Hence, the strains’ genotype, together with environmental factors during fermentation, determines to a large extent the presence of these compounds in the final product [9]. For example, *S. pastorianus* (employed in the production of *Pilsner*-style *lager*) [10,11] produces at low temperatures a subtle organoleptic profile mainly characterized by the presence of ester compounds such as ethyl acetate, ethyl hexanoate, and isoamyl acetate. On the other hand, *S. cerevisiae* (employed to produce ale *craft* beer fermented at high temperatures compared to lager) [10,11] produces a wider range of VCs like β-myrcene, ethyl octanoate, β-linalool, ethyl decanoate, octanoic acid, decanoic acid, ethyl acetate, and ethyl propionate, among others, providing a more complex organoleptic profile in beer [11,12]. The environmental stresses faced during the fermentation process, such as the presence of spoilage microorganisms, the fermentation temperature, nitrogen availability, and carbon sources in the wort, also impact the VC profile of the beer [2,7]. Therefore, depending on these variables, yeast cells produce different concentrations and types of VCs, allowing them to regulate their metabolism in terms of nitrogen uptake, membrane fluidity, and biomass generation [1,6].

The most relevant VCs produced by yeast are esters, phenols, and alcohols derived from the Ehrlich route [2,10]. However, most of the strains used in beer fermentation produce very similar and smooth organoleptic profiles, as found in many lager beers. While ale beers are marked by a broad array of esters and higher alcohols [1], lagers are generally characterized by a limited set of flavors and aromas [13]. For example, two commercial lager strains (Frisinga-TUM 3470 and Securitas-TUM 193) produced fewer esters and higher alcohols compared to a set of ale strains [14]. Interestingly, this set of ale strains showed different VC production profiles, demonstrating a pervasive intra-specific phenotypic variability among ale strains and a considerable impact on the final fermented product [14]. The lack of a wide range of VCs in lager styles is mostly explained by the low genetic diversity among *S. pastorianus* strains, strongly limiting the variety of lager styles. Consequently, the identification of new strains able to provide unique organoleptic profiles is an interesting approximation to expand the repertoire of lager beers and address the demands for new beer styles with different alcohol content and new sensory profiles [15,16].

A novel wild yeast with the capacity to be used in the brewing industry is *S. eubayanus*. The isolation of this cryotolerant yeast was initially reported by Libkind et al., 2011 [17] from *Nothofagus* forests in Argentina, and since then, it has been found in North America [18,19], East Asia [20], New Zealand [21], and, most recently, in Chile [22]. Interestingly, recent reports have shown considerable diversity of *S. eubayanus* in Patagonia [19,22]. Moreover, Patagonian strains exhibited significantly different fermentative profiles depending on the latitude from where the strains were obtained, in that isolates from northern Patagonian populations showed a better fermentative performance compared to isolates obtained from southern Patagonia and North American populations. However, in general, *S. eubayanus* attenuation levels were lower compared to the *S. pastorianus* W34/70 commercial strain, yet some strains still showed commercially accepted attenuation levels [22]. In this context, *S. eubayanus* has caught the interest of researchers and beer producers given the novel fermentative properties, such as fermentation at low temperatures (12 °C), efficient maltose utilization, and production of VCs (e.g., volatile-esters) that are pleasant in the final product [6]. Despite these attractive traits, the phenotypic diversity associated with the production of VCs among *S. eubayanus* isolates in wort fermentation has not been thoroughly characterized, constraining the potential utilization and knowledge of these yeasts. Furthermore, VC production in the *S. eubayanus* type strain has only been performed using HS-GC-FID (headspace gas chromatography with flame-ionization detector), where few VCs were quantified using internal standards [6,23]. Alternative approaches, such as HS-SPME-GC-MS (headspace solid-phase microextraction followed by gas chromatography-mass spectrometry), allows identifying a wider set of VCs whose identity is unknown before analysis. Furthermore, using this approach, relative ratios between samples can be compared, representing an alternative for brewing to identify potential novel aromas and flavors in beer.

In this study, we sought to investigate and characterize for the first time the fermentative and VC profile of different *S. eubayanus* strains from different Patagonian localities under fermentative conditions at low temperature (12 °C). To accomplish this, micro-fermentations were screened using the headspace solid-phase microextraction followed by gas chromatography-mass spectrometry method (HS-SPME-GC-MS) as a sensitive analytical means to identify VCs. The data gathered here demonstrate that a broad array of VCs is produced by wild *S. eubayanus* yeasts and highlights the potential of new strains for lager beer fermentation.

## 2. Materials and Methods

### 2.1. Yeast Strains Used in this Study

The *S. eubayanus* strains used in this study were obtained from bark samples from *Nothofagus pumilio* trees (lenga) collected in 10 different localities in Chile. All the strains were previously identified and described by Nespolo et al., 2020 [22] (Table S1). Briefly, strains were isolated from approximately 1 g of bark samples and immediately incubated in a 15 mL tubes containing 10 mL of enrichment media. This media contained: 2% yeast nitrogen base, 1% raffinose, 2% peptone, and 8% ethanol [24]. Samples were incubated for two weeks at 20 °C without agitation and were subsequently vortexed and plated (5 μL) onto YPD agar (1% yeast extract, 2% peptone, 2% glucose, and 2% agar). Isolated colonies were stored in glycerol 20% *v/v* and stored at −80 °C in the Molecular Genetics Laboratory yeast collection at Universidad de Santiago de Chile. The strains are available upon request.

### 2.2. Micro-Fermentation Assay

Micro-fermentation assays were performed on two genetically different *S. eubayanus* isolates from each location (Table S1). The different fermentations were made in beer wort “Pilsner Connoisseur” (Muntons), which was sterilized at 100 °C for 25 min. An initial inoculum of 5 mL was prepared in 6 °P beer wort, where a single colony from each strain was used. The sample was incubated for 24 h at 20 °C in constant agitation at 150 rpm. Subsequently, the inoculum was transferred to 50 mL of 12 °P beer wort and kept at 20 °C with shaking at 150 rpm for another 24 h. The cell cultures obtained were centrifuged at 5000 rpm for 5 min and used to calculate the final cell concentration for use in each micro-fermentation, according to the formula described by White and Zainasheff [25]. The micro-fermentation assay was performed in triplicate in 50 mL of previously oxygenated (15 mg/L) 12 °P beer wort and supplemented with 0.3 ppm of ZnCl_2_. Airlocks with 30% of glycerol were used. The weight of the bottles was measured throughout the progress of the fermentation process, registering on an analytical balance the CO_2_ loss over time (g/L). The commercial strain *S. pastorianus* W34/70 (*Sp*.W34/70) was used as a fermentation positive control. Similarly, micro-fermentations at 12 °C followed the same procedure.

### 2.3. Ammonium and Amino Acid Analysis

Ammonium and Amino acid consumption was evaluated at different time-points during the fermentation process. For this, a 100 mm cannula was inserted into the rubber stopper to perform periodical sampling. Samples were obtained at 24, 48, and 168 h by extracting 0.5 to 1 mL of fermented beer wort. Each sample was centrifuged at 13,000 rpm for 5 min, and the supernatant was recovered and stored at −20 °C. Fermented wort was processed following the protocol described by Gomez-Alonzo et al. 2007. Briefly, 120 µL of the sample was incubated with 20 µL of diethylethoxymethylen-emalonate (DEEMM, SIGMA) for 24 h at room temperature in a solution containing 580 µL of a borate buffer (pH = 9) and 250 µL of absolute methanol (Fisher Chemical). The sample was heated at 70 °C for 2 h to allow the complete degradation of excess DEEMM and reagent byproducts. After the derivatization reaction, 20 µL of the processed samples were analyzed by High-Performance Liquid Chromatography (HPLC, Shimadzu) with a C18-HL column (250 mm × 4.6 mm), a binary gradient (phase A: phase B:), and a flow rate of 0.9 mL/min. For detection, a photodiode array detector (Shimadzu, Kokyo, Japan) set at 270, and 280 nm was used. The different compounds were identified according to the retention time and UV-vis spectral characteristics of the derivatives of the corresponding standards and were quantified using the internal standard’s method.

### 2.4. HS-SPME-GC-MS Analysis

Analysis of the production of all VCs was carried out by the combination of four analytical procedures: headspace, solid-phase microextraction, gas chromatography, and mass spectrometry (HS-SPME-GC-MS, Thermo Scientifics, Whaltam, MA, USA). A headspace vial was loaded with 2 mL of sample and equilibrated at extraction temperature (60 °C) for 30 min. Then, a 50/30 μm divinylbenzene/carboxenpolydimethylsiloxane fiber (DVB/Car/PDMS; Supelco, Bellefonte, PA, USA) was introduced within the headspace vial in order to extract the volatiles, through a silicon septum for 30 min. Targeted volatiles loaded in the fiber were analyzed by using a GC-MS (Thermo Scientifics Trace GC Ultra, equipped with Thermo Scientifics ISQ quadrupole mass spectrometer and autosampler Thermo Scientifics Triplus) and GC-MS/MS (Thermo Scientifics Trace 1300 GC, equipped with Thermo Scientifics TSQ Triple quadrupole mass spectrometer and autosampler Thermo Scientifics Triplus RHS) in full-scan mode. The desorption of the fiber was carried out (splitless mode) at 250 °C for 5 min and cleaning at 270 °C for 15 min. Helium was passed at a constant flow (1.2 mL/min) for serving as a carrier gas. Mass spectrometry detection was performed under electron impact (EI) ionization at 70 eV by operating in the full-scan acquisition mode in the 40–400 m/z range. Ion source and transfer line temperature were maintained at 250 °C, respectively. Potential emanations were analyzed using Xcalibur Software (Thermo Electron Corporation) matching mass spectra with those saved in the National Institute of Standards and Technology (NIST) MS Spectral Library 2014. Chromatographic peaks were considered “unknown” when their similarity index (SI) and reverse similarity index (RSI) were less than 850 and discarded in this identification process. These parameters refer to the degree at which the target spectrum matches the standard spectrum in the NIST Library (a value of 1000 indicates a perfect fit). Selected chromatographic peaks were checked with their respective chemical standards and Kovats indexes [26].

### 2.5. Statistical Analyses

All statistical analyses were performed using biological triplicates. One-way ANOVAs (Analysis of Variance) and Pearson non-parametric correlations (*t*-Test) were performed using GraphPad Prism 8.01 for Windows, GraphPad Software, La Jolla, CA, USA, www.graphpad.com. The differences were considered statistically significant at *p*-values < 0.05. A principal component analysis (PCA) was performed using R software [27], and the “prcomp” package stats 3.6.0 and plotted using the “ggbiplot” package. Temperature data for the correlation analyses was obtained from the Center of Climate and Resilience Research (http://www.cr2.cl/datos-de-temperatura/). The data obtained correspond to measurements of daily average temperature recorded in 33 stations of the Chilean Meteorological Center and in 196 stations of the General Direction for Water during 2018.

## 3. Results

### 3.1. Differences in Fermentation Capacity Across S. eubayanus Isolates

**Figure 1:**
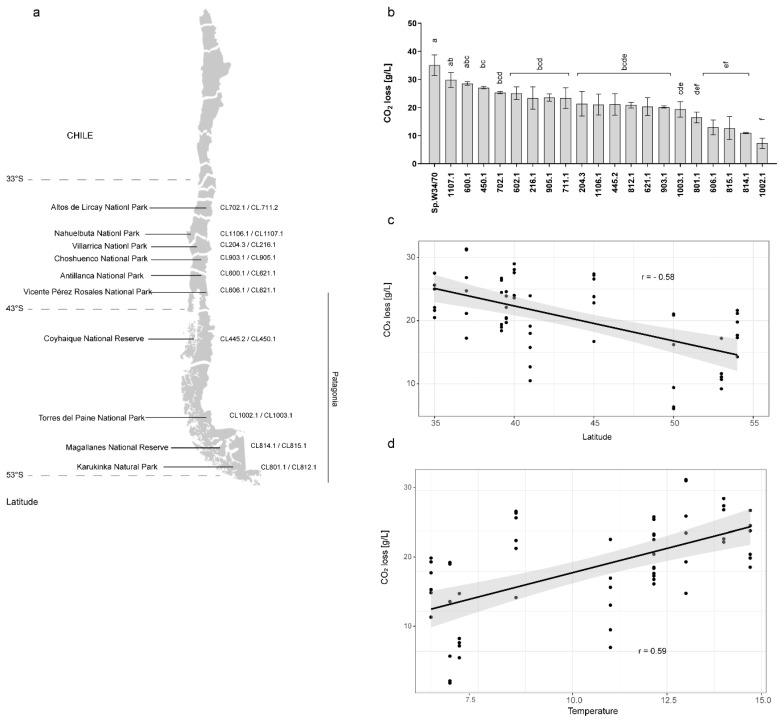
*Saccharomyces eubayanus* fermentation performance in lager wort. (**a**) The geographical location of the sampling sites and the strain codes from each locality. (**b**) Total CO_2_ loss [g/L] in fermentations carried out by *S. eubayanus* strains. Lager strain *S. pastorianus* W34/70 was used as a fermentation control. Different letters reflect statistically significant differences between strains with a *p*-value < 0.05, one-way ANOVA. (**c**) Latitude and (**d**) annual average temperature at the isolation site of each strain vs. CO_2_ loss (Pearson Correlation). Each dot represents a fermentation replicate. Three biological replicates were analyzed per strain.

To evaluate the fermentative capacity and differences in kinetic profiles of *S. eubayanus* strains when brewing wort, we performed a micro-fermentation assay using 20 strains obtained from different sites in central and Chilean Patagonia [22] (Figure 1a, Table S1). For this, strains were inoculated in beer wort, and CO_2_ release was measured for 14 days (see Methods). The strains exhibiting the greatest levels of CO_2_ release at the end of the fermentation were CL1107.1 (Nahuelbuta National Park, *p*-value = 0.422, ANOVA) and CL600.1 (Antillanca National Park, *p*-value = 0.294, ANOVA), neither of them exhibited significant differences when compared to the commercial strain (*Sp.*W34/70) (Figure 1b). Interestingly, these two strains were obtained from different sampling sites (central Chile and northern Chilean Patagonia, Figure 1a, and Figure S1). On the other hand, strains with the lowest fermentative capacity were CL1002.1 (Torres del Paine National Park, *p*-value < 0.0001, ANOVA), CL814.1 (Magallanes National Reserve, *p*-value < 0.0001, ANOVA), CL815.1 (Magallanes National Park, *p*-value < 0.0001), CL606.1 (Vicente Pérez Rosales National Park, *p*-value < 0.0001, ANOVA), and CL801.1 (Karukinka Natural Park, *p*-value < 0.0001, ANOVA), all of them from the central and southern Chilean Patagonia sampling sites (Figure 1a,b and Figure S1).

To determine whether fermentation performance correlated with climate and/or geographic conditions, we performed a correlation test between CO_2_ release and: i) latitude and ii) average yearly temperature. We found a significant correlation for both parameters: latitude (Pearson r = −0.58, *p*-value < 0.001, *t*-Test) and average yearly temperature per isolation locality (Pearson r = 0.59, *p*-value < 0.001, *t*-Test) vs. CO_2_ loss (Figure 1c,d). The observed differences could correlate with fermentation capacity and CO_2_ release, indicating different fermentative potentials between strains depending on their geographic origin.

### 3.2. Volatile Compound Production in S. eubayanus Isolates

**Figure 2:**
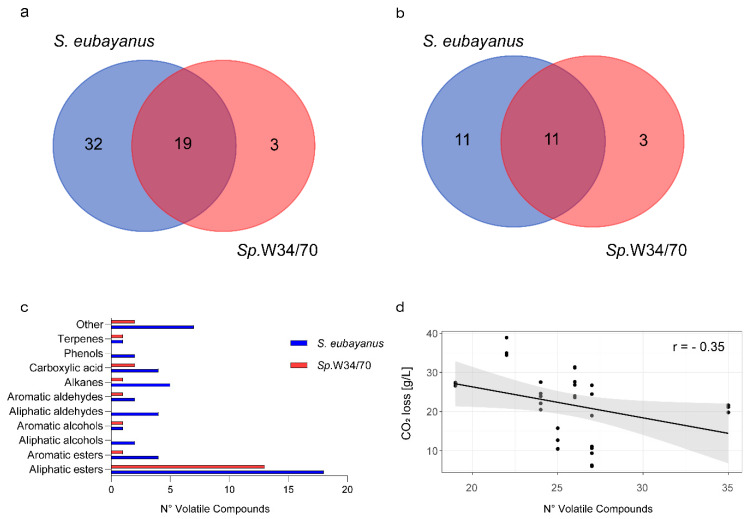
Volatile compounds identified in lager beer micro-fermentations using wild *S. eubayanus* and the *Sp*.W34/70 strain. (**a**) Total VCs identified in beer wort fermented by wild Chilean *S. eubayanus* (Blue) strains and *S. pastorianus* W34/70 (Red). (**b**) Total esters identified in beer wort fermented with wild Chilean *S. eubayanus* (Blue) strains and *S. pastorianus* W34/70 (Red). (**c**). Main chemical groups detected in fermented beer wort. (**d**) Correlation between the number of VCs produced and fermentative capacity. Each point depicts a biological replicate.

To characterize the VC production profiles of the *S. eubayanus* strains, we analyzed the fermented wort by HS-SPME-GC-MS VCs and identified VCs whose identity is unknown, rather than using internal standards for commonly known compounds. To attain this, we selected 10 strains based on the two following criteria: (i) One representative isolate from each sampling site and (ii) significantly different fermentative capacities (CO_2_ release levels) between strains to maximize the genetic and phenotypic diversity. Therefore, we selected strains with high fermentative capacity (CL1107.1 and CL450.1), mid-fermentation capacity (CL711.2, CL216.1, CL905.1, CL602.1, and CL812.1), and low fermentative capacity (CL606.1, CL1002.1, and CL814.1) (Figure 1b). HS-SPME-GC-MS analyses using the 10 selected strains, together with the *Sp.*W34/70 lager control, allowed the identification of 55 different compounds in all fermented wort (Figure 2a, Table S2). Out of these compounds, 52 were found in *S. eubayanus* strains and 22 were found in the lager strain (*Sp.*W34/70) (Figure 2a). Interestingly, 32 compounds were exclusive to *S. eubayanus* and were not detected in *Sp.*W34/70. In comparison, only three compounds (ethyl hexanoate, ethyl heptanoate, and ethyl dihydrocinnamate) were found solely in the commercial strain *Sp*.W34/70, resulting in 19 compounds common to both species (Figure 2a, Table S2). These results demonstrate that all compounds (except ethyl hexanoate, ethyl heptanoate, and ethyl dihydrocinnamate) generated by the lager strain are found in at least one wild isolate. Among all the analyzed compounds, esters represent the main fraction of the VCs identified (26 in wild *S. eubayanus* and 15 in *Sp*.W34/70) (Figure 2b,c), where 14 compounds were found in common across both species (Figure 2b). Interestingly, of the whole set of VCs identified, only a single higher alcohol, phenethyl alcohol, was detected by HS-SPME-GC-MS. In contrast, no phenolic compounds were identified in the lager strain. However, in wild *S. eubayanus,* we discovered two different phenolic compounds: 4-vinylguaiacol (4-VG), which was found in all strains and 2,4-Di-tert-butylphenol, which was only identified in CL1002.1 and CL602.1. A negative and significant correlation was found between the number of VCs produced at the end of the fermentation process and fermentation capacity (Pearson r = −0.35, *p*-value < 0.05, *t*-Test, Pearson Correlation). In this context, strains with a higher fermentative capacity produced a lower number of (identified) compounds, suggesting that the production of a wider number of VCs is not directly related to high fermentation capacity (Figure 2d).

### 3.3. Main Volatile Compounds Identified in Fermented Wort

**Figure 3:**
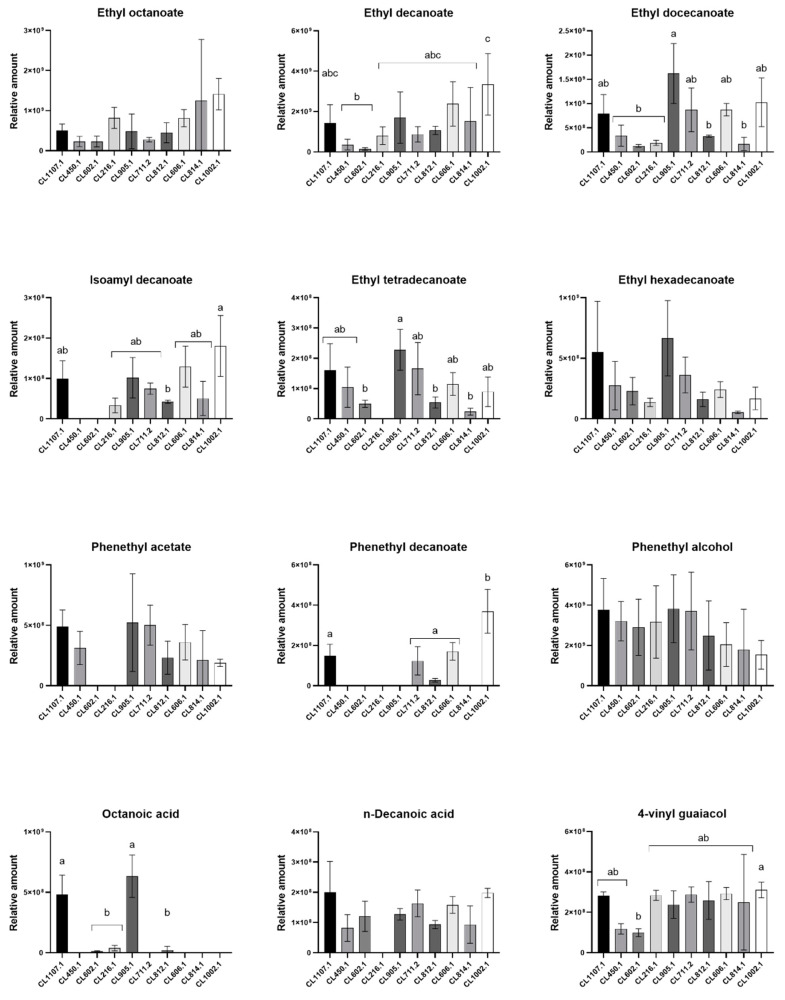
Relative amounts of the main volatile compounds in fermented wort at 12 °C. The strains are distributed according to their fermentative capacity from high to low levels (left to right). Different letters reflect statistically significant differences between strains with a *p*-value < 0.001, one-way ANOVA. No-letters strains do not show statistically significant differences.

To improve our comparative analysis of VC production profiles across strains, we selected compounds comprising the highest relative amounts, representing at least 45% of the total VC area detected and that were identified in at least five *S. eubayanus* strains (Table 1, Figure 3). In this way, we selected 12 compounds, most of them aliphatic esters. Of these, ethyl octanoate (aliphatic ester), ethyl hexadecanoate (aliphatic ester), phenethyl alcohol (aromatic alcohol), and n-decanoic acid (carboxylic acid) were found in all strains in similar relative amounts (Figure 3). On the other hand, among the aliphatic esters detected by HS-SPME-GC-MS, ethyl decanoate was the most abundant VC, especially in the CL1002.1 isolate from Torres del Paine National Park (3300 ± 879.8, *p*-value < 0.05, ANOVA) (Table 1). Other compounds, such as isoamyl decanoate, phenethyl acetate (aromatic ester), phenethyl decanoate (aromatic ester), and octanoic acid (carboxylic acid) (Figure 3), were exclusively detected in a subset of strains (Table 1). Within the detected off-flavors, 4-VG (phenols) exhibited one of the highest area contributions to the profiles of the strains, being identified at similar levels in all strains (*p*-value > 0.05, ANOVA, Figure 3), except for CL602.1 (*p*-value < 0.05, ANOVA), exhibiting a lower detected relative amount compared to the other strains (98.8 ± 11.4).

**Figure 4:**
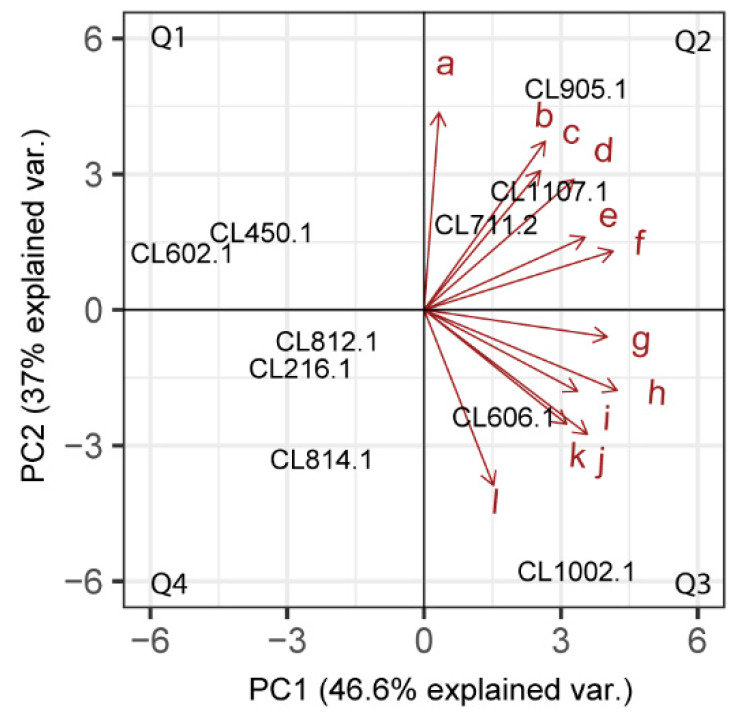
Principal Component Analysis of VC production across *S. eubayanus* strains. Distribution of the selected strains based on the leading VCs produced. a: Phenethyl Alcohol, b: Ethyl hexadecanoate, c: Octanoic acid, d: Ethyl tetradecanoate, e: Phenethyl acetate, f: Ethyl dodecanoate, g: n-Decanoic acid, h: Isoamyl decanoate, i: 4-vinyl guaiacol, j: Ethyl decanoate, k: Phenethyl decanoate, l: Ethyl octanoate. The strains were distributed in four quadrants, Q1: First group. Q2: Second group. Q3: Third group. Q4: Fourth group.

To reduce the dimensionality of our dataset and interpret the profile of VC production for each strain, we performed a global principal component analysis (PCA) using the 12 selected VCs (Figure 4)**.** The two first component explained 46.6% and 37% of the observed variation, respectively, allowing us to classify the *S. eubayanus* strain profiles in four groups: (Q1) CL450.1 and CL602.1, (Q2) CL812.1, CL216.1 and CL814.1, (Q3) CL905.1, CL1107.1 and CL711.2, and (Q4) CL606.1 and CL1002.1. Interestingly, strains in Q2 produced higher amounts of VCs, such as phenethyl alcohol and ethyl tetradecanoate, while those in Q3 showed higher areas for isoamyl decanoate and ethyl decanoate. This analysis suggests that strains with similar aromatic profiles do not have a common geographical or genetic origin, with isolates from central and southern Chile being found in all groups. Similarly, except for phenethyl alcohol (r = −0.75, *p*-value < 0.05, *t*-Test, Pearson Correlation), no significant correlation was found (*p*-value> 0.05, *t*-Test, Pearson Correlation) when relating latitude (origin of the strains) with the relative amount of the primary VC produced (Figure S2). Altogether, our results show the broad spectrum of VC profiles in *S. eubayanus* strains from different sites, and highlight the organoleptic potential for innovation in the brewing industry of this newly discovered yeast.

### 3.4. Fermentation Temperature Impacts the Volatile Compound Profile in S. eubayanus Strains

Depending on the strain and fermentation temperature, producers can fine-tune the aromatic profiles of beverages. We compared the temperature dependence of *S. eubayanus* strains and their impact on the production of VCs in brewing wort at the end of fermentation. For this, we selected two strains (CL905.1 and CL602.1) with (i) similar fermentative capacity (Figure 1b) and (ii) different aromatic profiles and located in different quadrants in the PCA (Figure 4). In this way, we performed new fermentation batches at 12 °C and 20 °C and identify the VCs produced at both temperatures using HS-SPME-GC-MS. Fermentative profiles were similar between strains at 20 °C and did not show statistically significant differences for fermentation CO_2_ release. At 12 °C, the strains show statistically significant differences for fermentation CO_2_ release with a *p*-value < 0.001, ANOVA (Figure S3). We found that VC profiles differed in a strain- and temperature-dependent manner. Notably, at 12 °C, the two isolates produced 16 compounds in common, rising to 20 when fermentation was run at 20 °C (Table S3). In strain CL905.1, we identified 23 and 32 compounds at 12 °C and 20 °C, respectively, while the opposite was observed in CL602.1, where we identified 24 and 21 VCs at 12 °C and 20 °C, respectively. In the case of CL602.1, the main chemical group affected by temperature was the aliphatic esters, while in CL905.1, the main chemical groups with more compounds detected at higher temperature were aliphatic esters (1-hexanol, 2-ethyl, propanoic acid, 2-methyl,3-hydroxy-2,2,4-trimethylpentyl ester and ethyl 9-hexadecanoate), aliphatic alcohols (linalool, 1-octanol and 1-decanol), aromatic aldehydes (benzaldehyde, benzeneacetaldehyde and benzaldehyde, 4-methyl), and other compounds (1,1,3-trimethyl-3-phenylindane) (Table S3). These results demonstrate that fermentation temperature differentially modulates the total number of VCs depending on the strain and genetic background, likely impacting the aromatic profiles of the beers produced (Table 2, Table S3).

**Figure 5:**
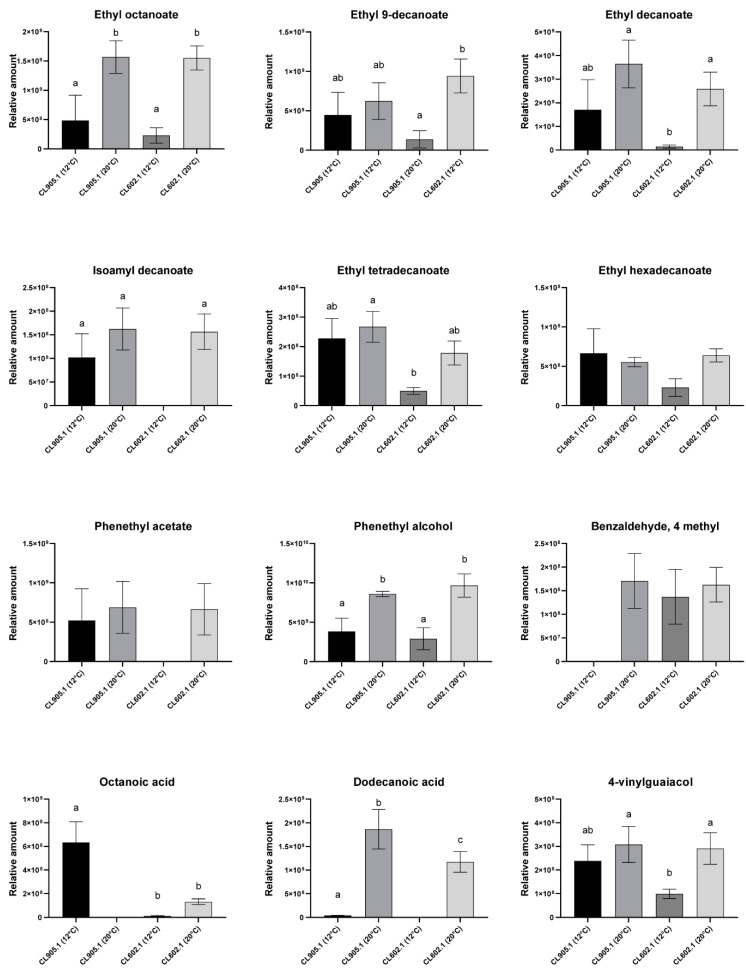
Relative amounts of main volatile compounds identified in fermented wort at 12 and 20 °C. CL905.1 (Choshuenco National Park) and CL602.1 (Antillanca National Park) were fermented at 12 °C and 20 °C, respectively. Different letters reflect statistically significant differences between strains with a *p*-value < 0.001, one-way ANOVA. No-letters strains do not show statistically significant differences.

We next selected the compounds with the highest area percentage (representing approximately 65% of the area of the whole peak) in CL905.1 and CL602.1 strains (Figure 5, Table 2). In this way, we identified 12 main compounds at 12 °C and 20 °C, for which relative areas were compared between both strains. As previously mentioned, not only the number of VCs changed with temperature, but we also found differences in the relative amount produced depending on the fermentation temperature. For example, ethyl octanoate (aliphatic ester) and phenethyl alcohol (aromatic alcohol, higher alcohol) (Figure 5, Table 2) significantly increased at 20 °C (*p*-value > 0.001, ANOVA), independently of the strain. On the other hand, higher levels in the relative amount of 4-VG at 20 °C compared to 12 °C was only observed in CL602.1 (*p*-value > 0.001, ANOVA) and not in CL905.1 (Figure 5). Similarly, other ester compounds significantly increased with temperature solely in CL602.1, such as: ethyl 9-decanoate, ethyl decanoate, isoamyl decanoate, and phenethyl acetate (*p*-value > 0.001, ANOVA). Depending on the strain, a higher fermentation temperature allowed us to find novel compounds undetected at 12 °C. For example, benzaldehyde 4-methyl (aromatic aldehyde) was exclusively detected in CL905.1 at 20 °C, but not at 12 °C. In contrast, in CL602.1, this compound was detected at both temperatures (*p*-value < 0.001, ANOVA). These results demonstrate that fermentation temperature results in different profiles in both strains in terms of the number of compounds, but also in their relative amounts, representing a strain × temperature specific interaction.

### 3.5. Nitrogen Consumption Differentially Impacts the Prodcution of Volatile Compounds

**Figure 6:**
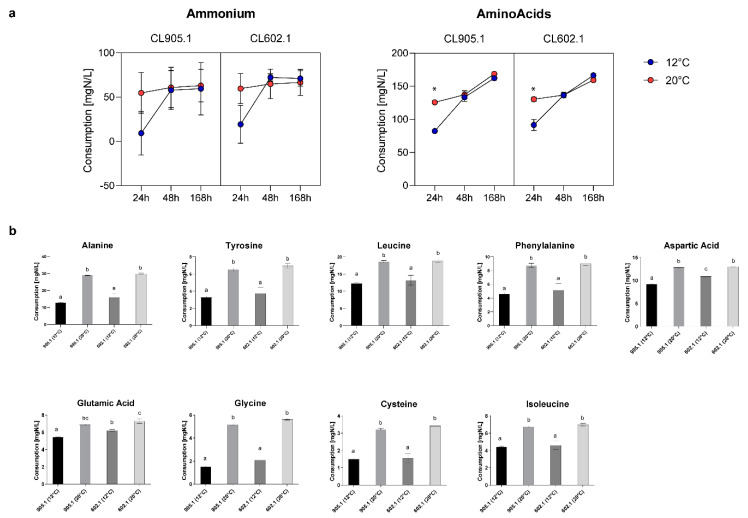
Differential yeast assimilable nitrogen (YAN) consumption in CL905.1 and CL602.1 strains at 12 °C and 20 °C. (**a**) Ammonium and amino acid consumption kinetics at 24, 48, and 168 h. Blue dots: 12 °C fermentation; Red dots: 20 °C fermentation. (*) depict significant differences between temperatures with a *p*-value < 0.001, one-way ANOVA. (**b**) Main differences in amino Acid consumption between temperatures. Different letters depict significant differences between strains with a *p*-value < 0.05, one-way ANOVA.

The ability to assimilate different nitrogen sources in the brewing wort directly impacts the physiology of yeasts during the fermentation process. Simultaneously, the differential consumption of nitrogen sources could affect the aroma and flavor profiles of fermented wort [1,28,29,30]. In this context, to determine the nitrogen consumption profiles of the two strains (CL905.1 and CL602.1) at 12 °C and 20 °C during fermentation, yeast assimilable nitrogen (YAN) consumption was measured by HPLC after 24, 48, and 168 h of fermentation (Figure 6). In the case of ammonium, that represents 29.02% (Table S4) of the overall YAN in the beer wort, 21.98% ± 1.31% of all the available ammonium was consumed by 24 h at 20 °C, while at 12 °C, a similar fraction (14.82% ± 10.50%, *p*-value < 0.001, one-way ANOVA) was reached at 48 h (Figure 6a, Table S4). In this way, when we compared the effect of temperature over the consumption of ammonium in both strains, significant differences were observed only at 24 h (*p*-value < 0.05, ANOVA) (Figure 6a), while at 168 h, most of the ammonium was already consumed in both strains, suggesting a temperature dependent (and not strain dependent) nitrogen consumption rate.

**Figure 7:**
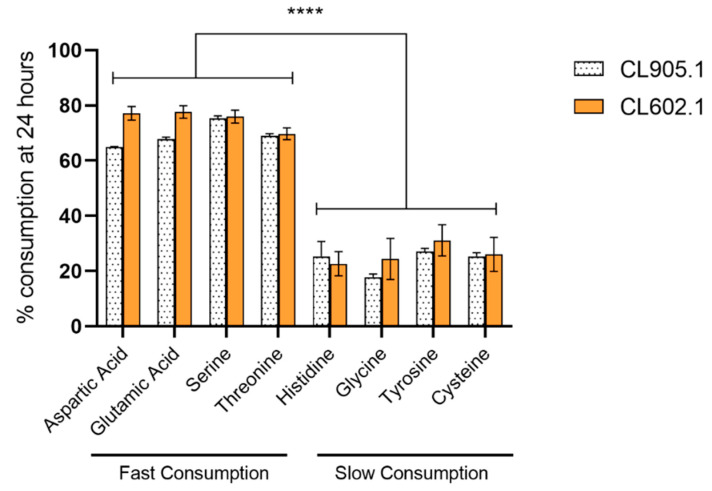
Preferred amino acid consumption in *S. eubayanus* strains at 24 h. Gray: CL905.1; Orange: CL602.1. (****) depict significant differences between amino acids with a *p*-value < 0.001, one-way ANOVA.

Subsequently, we analyzed the consumption of amino acids in wort. In this way, we found that their consumption rate was slower compared to ammonium, where both strains exhibited significant differences at 24 h between 12 °C and 20 °C (Figure 6a, Table S4). Strain CL905.1 consumed 82.44 ± 1.95 mg N/L at 12 °C, while a higher value was reached (125.77 ± 2.13 mg N/L, *p*-value < 0.001, ANOVA) when the fermentation temperature was set to 20 °C. Similarly, CL602.1 showed an increase in amino acid consumption between 12 °C (91.56 ± 8.41 mg N/L) and 20 °C (130.36 ± 3.28 mg N/L, *p*-value < 0.001, ANOVA) (Table S4), demonstrating differential amino acid consumption depending solely on the fermentation temperature, but not on these genetic backgrounds. Of all the amino acids analyzed in this study (Table S5), solely glutamic acid and aspartic acid significantly differed at 12 °C (Figure 6b) (*p*-value <0.05, ANOVA) between strains, while for the rest of the amino acids, the differences were due to an increase in fermentation temperature, independently of the strain (Figure 6b). Additionally, we determined that, for both strains, several amino acids, such as aspartic acid, glutamic acid, serine, and threonine, were rapidly consumed in the wort (>60%) at 24 h, demonstrating the preference of *S. eubayanus* strains for these nitrogen sources (Figure 7, Table S5). On the other hand, amino acids such as histidine, glycine, tyrosine, and cysteine had lower consumption levels (<30%) at 24 h (low preference) and were efficiently consumed (>80%) only at 168 h (Table S7).

## 4. Discussion

Beer is a complex mixture of ingredients, brewed from raw materials including water, malt, hops, and yeast, that contains a broad range of different components that react and interact at all stages of the brewing process [2]. Yeasts employed in industrial fermentation processes can convert relatively high concentrations of sugars into ethanol, carbon dioxide, and a wide range of secondary metabolites [31], which impact in several ways the aromatic profile of beer. While these compounds are only produced at low concentrations, they are responsible for the complex aromas of fermented beverages. Currently, alternative yeast strains are being considered to provide new and innovative beers [32]. Under this scenario, and considering the recent market trends, bioprospecting research on pioneering wild yeast strains with the capacity to produce new beers is becoming an attractive field in food research. In this way, the recent finding of *S. eubayanus* in Patagonia is a crucial element for beer microbial innovation [6,9,17,22]. In this study, we analyzed the fermentation capacity and VC production of a genetically diverse set of Patagonian *S. eubayanus* strains from Chile, previously described by Nespolo et al. [22]. To our knowledge, this represents the first study that explores the landscape of VCs produced during beer fermentation in *S. eubayanus* using HS-SPME-GC-MS. The differences observed between these strains relate to those previously reported, where strains with higher stress tolerance developed a greater fermentative capacity than other strains [22,33]. Accordingly, the worldwide increase in beer consumption and the continuous growth of microbrewers highlight the need for the characterization of the aromatic profile of novel beers, requiring tools to evaluate beer quality and authenticity. In this context, the HS-SPME-GC-MS method, used in this analysis, has been reported as a fast and sensitive technique for the identification of VCs in both industrial and craft beers [10,34]. This technique develops reproducible data, with the improvement that avoids the injection of the standards, favoring several advantages, such as the time and cost of the analysis [10]. In HS-SPME (headspace solid-phase microextraction), analytes are extracted into a thin fused-silica fiber coated with the extracting phase by immersing it in the headspace of solid or liquid samples. Then, by GC-MS, compound identification is based on mass spectra matching with the standard NIST 08 MS library and on the comparison of retention indices (RI) sourced from the NIST Standard Reference Database MS Spectral Library 2014. The identification of the VCs by comparing the experimental mass spectra based on spectral similarity with those stored in the NIST MS library database, without chemical reference standards, should be regarded as putative compounds identifications (Level 2). Moreover, the determination of Kovats retention indices (RI) and their match with those available in previous literature [10,34] represents a useful tool for identification purposes, being independent of the operating conditions, except for the polarity of the used stationary phase. Furthermore, considering complex matrices as wort fermentation, which can contain hundreds of VCs to be analyzed, this analytical approach described above has the advantage that avoids the injection of the pure standards, favoring the time and cost of the analysis and the limitation that several standards are not commercially available.

In this study, we compared the VC production profile of two different yeast species, *S. pastorianus* (*Sp.*W34/70, commercial lager strain) and *S. eubayanus* (wild isolates from Chilean Patagonia) using HS-SPME-GC-MS. The main differences between *S. eubayanus* and *S. pastorianus* lie in the number (51 vs. 22) and the relative amounts of esters and phenols identified. These findings reflect the aromatic profile of the commercial strain, which likely lost the ability to produce a broad array of VCs due to a complex brewing domestication process. During this process, the fermentative capacity of the strain was prioritized over the production of complex aromatic profiles [35]. Esters were found in a higher proportion in the two yeast species analyzed in our study. These compounds are produced during the fermentation process and are usually characteristic of young beers [10]. Moreover, other compounds were identified, such as higher alcohols and phenols, of which the production is influenced by several factors, such as wort composition, fermentation parameters, and beer maturation state [34]. The presence of these compounds, together with volatile-esters, is responsible for contributing to the final beer flavor [1,8]. One of the most distinctive VCs produced by wild yeast is 4-VG [8]. The aroma and flavor descriptors used for this phenolic compound (identified in all *S. eubayanus* stains) are wide-ranging, and include stable, barnyard, horsey, leathery, smoky, spicy, clove, medicinal, and others (Dzialo M. et al. 2017; Lentz M. 2018), contributing significantly to the organoleptic profile. The role of phenols in the aromatic profile of the beer is questionable; in individual beer styles, such as Bavarian wheat beers and Belgian white beers (Lentz M. 2018), the phenolic flavors are desired and contribute to the style of the beer. However, the same VCs are perceived as undesirable in other fermented beverages and are commonly referred to as “phenolic off-flavors” (POF). Similar studies using other *S. eubayanus* strains [36], indicated that this species produces higher levels of esters, phenols, and higher alcohols in fermentations at low temperatures (15 °C), compared to *Sp.*W34/70. Indeed, our results followed the same trend, as we detected an additional set of VCs previously uncharacterized in lager beers. In this context, considering the great and novel diversity of VCs produced, the *S. eubayanus* strains considered in our study show an innovative potential for alternative beer production.

The volatile fraction of beer includes over 800 different compounds, but only several dozen of these are considered flavor active [37]. In this context, we selected compounds that could be relevant to discriminate the set of *S. eubayanus* strains considered in our study and performed a PCA. When analyzing the distribution of the isolates, according to their aromatic profile, we observed a random distribution of the *S. eubayanus* strains. There are no strains grouped by fermentation capacity or geographic isolation zone. By dividing the PCA into four quadrants (Q1–Q4), it is remarkable that strains with similar fermentative profiles are distributed in opposite quadrants. Given this, the quantity and variety of the VCs produced by *S. eubayanus* isolates seem to be strain-specific traits, and reflect the great genetic diversity present in this species, as reported previously [19,22]. In this context, the genetic diversity and the different VCs identified in *S. eubayanus* resemble those described for *S. cerevisiae*, rather than *S. pastorianus*, likely determined by the genetic diversity of the species. *S. eubayanus* is not the sole species to produce large amounts of volatile esters. For example, *S. cerevisiae* strain Safale S-04 (Belgium) produces a wide range of these compounds, such as: ethyl acetate, isoamyl acetate, phenethyl acetate, ethyl decanoate, ethyl hexanoate, and ethyl octanoate [38,39], responsible for giving the fermented wort a fruity profile and in some cases a mixture with solvent aromas [7]. These profiles are attractive in the worldwide beer market, and the development of lager beers with fruity aromatic profiles fermented at low temperatures could likely be of interest in the global beer industry.

The parameters that affect the response of yeasts during the biosynthesis of aromatic compounds are diverse [7]. The production of active aromatic compounds is directly related to the yeast strain used in fermentation [1], as the genome of each strain is unique and will ultimately impact the final aroma profile of the beverage [40]. This is very well reported in *S. cerevisiae* [12,41,42,43], while in *S. eubayanus*, only a handful of studies have evaluated the aromatic profile in beer [6,23,36]. Other relevant parameters reported are wort composition, oxygen [44], yeast assimilable nitrogen (YAN) [11,45,46], and temperature of fermentation [7,47,48]. In the present study, it was possible to observe that temperature exerted an effect on the total production of VCs depending on the genetic background of the *S. eubayanus* strains. These changes have been previously reported in yeast of the genus *Saccharomyces*, where fermentation temperatures above 20 °C modified the production of VCs in beer and wine [47]. A special case was the POF 4-VG, where no changes were observed in strain CL905.1, while in CL602.1, its content significantly increased when the fermentation temperature was increased (12° to 20 °C). Most reports focused on the study of 4-VG have set out to find ways to reduce their production within the fermentation process by modulating precursors in the wort [8]; however, our results also demonstrate the impact of strain × temperature on the production of 4-VG. From a physiological perspective, none of the parameters analyzed in this study may solely explain the changes in the VCs produced in *S. eubayanus* strains, suggesting a complex temperature × strain response under fermentative conditions. An example is the YAN content. During alcoholic fermentation, the cells import and metabolize the YAN in the wort together with other nutrients to produce biomass as well as VCs [1,30,49,50]. Therefore, depending on the amount and type of assimilated YAN, different VCs will be generated, thus determining the aromatic profiles of the fermented wort [7,50]. The YAN content can be separated into two groups, ammonium and amino acids. Ammonium is the primary nitrogen source in beer and wine yeasts [30]. Nitrogen consumption has been widely studied in wine yeast, establishing hierarchies in nitrogen source utilization [29]. For example, ammonium, glutamine, glutamate, and asparagine are considered good or preferred nitrogen sources. In *S. eubayanus*, we found that ammonium, aspartic acid, glutamic acid, serine, and threonine nitrogen sources were rapidly consumed and may represent the preferred nitrogen sources for wort fermentation. Interestingly, no differences were observed between strains in terms of such preferences, although temperature significantly impacted the nitrogen consumption rate at early timepoints.

## 5. Conclusions

Sensory properties of beer are influenced by the wide variety of VCs produced by yeast. The Patagonian populations of *S. eubayanus* comprise phenotypically distinct individuals, representing a niche of innovation for a wide range of volatile compounds in beer. Overall, *S. eubayanus* strains produce a more significant number of volatile compounds compared to a commercially available lager yeast. In this context, fermentation temperature plays an essential role, together with the genetic background of the *S. eubayanus* strain, in determining the VC profile of the resulting beer. The variation of the fermentation temperature can increase (CL905.1) or decrease (CL602.1) the amount and type of VCs depending on the strain, generating a fine-tuning mechanism on the total production of compounds that contribute to the complete and complex aromatic profile of the beer. HS-SPME-GC-MS is a handy tool to analyze the main VCs present in the fermented wort, allowing the reliable identification of the large variety of compounds produced during the fermentation process. In this way, the procedures that were followed in this report lay the foundation to study a wider set of strains, deepening our understanding of the catalog of VCs produced by *S. eubayanus* natural isolates, which have an enormous innovation potential.

## Figures and Tables

**Table 1:** Main Volatile Compounds detected in *S. eubayanus*.

			Relative Amount (×10^6^) and SEM									
			Strains									
Chemical Group	Compound Name	RI	CL1107.1	CL1002.1	CL905.1	CL814.1	CL812.1	CL711.2	CL606.1	CL602.1	CL450.1	CL216.1
**Aliphatic esters**	Ethyl octanoate	1192.64	506.1 ± 91.8	1400 ± 226.4	483.1 ± 250.2	1300 ± 1100	446.4 ± 144	276.7 ± 31.7	812.9 ± 123.5	228.8 ± 76	230.2 ± 73.7	819.5 ± 151.7
	Ethyl decanoate	1391.77	1400 ± 522.4	3300 ± 879.8	1700 ± 734.6	1500 ± 1200	1100 ± 118.7	870.3 ± 219.4	2400 ± 635.3	141.7 ± 38.3	362 ± 152.8	802.2 ± 253.5
	Ethyl dodecanoate	1443.83	793.6 ± 226	1000 ± 289.6	1600 ± 357.4	164.5 ± 96.9	325.8 ± 11.7	868.5 ± 260.4	872.2 ± 73.8	123.7 ± 17	335.3 ± 125.3	186 ± 30.2
	Isoamyl decanoate	1590.63	99.7 ± 25.7	180.5 ± 43.5	102.1 ± 28.9	50.8 ± 30	42.4 ± 2.1	74.9 ± 8.1	129.4 ± 29.3	N/D	N/D	33.5 ± 10.5
	Ethyl tetradecanoate	1790.87	160.3 ± 50.9	89.8 ± 28.2	228.2 ± 38.9	23.7 ± 8	54.3 ± 10.5	166 ± 50	115.2 ± 21.6	49.7 ± 6.8	104.5 ± 38.3	N/D
	Ethyl hexadecanoate	1991.92	552 ± 241.4	169 ± 53.7	666 ± 179.7	53.4 ± 7.4	161.3 ± 34.4	363 ± 85.2	242.5 ± 37.5	229.4 ± 65.1	274.3 ± 115.2	136.9 ± 20
**Aromatic esters**	Phenethyl acetate	1250.12	490.8 ± 79.1	189.8 ± 17	522.4 ± 233.1	212.9 ± 172.2	232.1 ± 78.9	501.7 ± 95.5	360.2 ± 84.7	N/D	N/D	312.6 ± 79.2
	Phenethyl decanoate	2062.65	149.5 ± 32.8	369.7 ± 62.8	N/D	N/D	27.7 ± 5.3	123.7 ± 40.7	170.8 ± 25.3	N/D	N/D	N/D
**Aromatic alcohol**	Phenethyl Alcohol	1116.19	2900 ± 1600	1500 ± 409.3	3800 ± 971.5	1800 ± 1400	2500 ± 992.4	3700 ± 1100	2000 ± 627	2900 ± 805	3200 ± 565.2	3200 ± 1000
**Carboxylic acid**	Octanoic acid	1171.39	483 ± 91.8	N/D	633.5 ± 101.3	N/D	22.9 ± 17.4	N/D	N/D	12.5 ± 2.4	N/D	39 ± 13.1
	n-Decanoic acid	1360.8	200 ± 59	198 ± 8.8	127.5 ± 11	93.3 ± 43.8	93.4 ± 7.8	163.3 ± 25.6	158.3 ± 16.1	N/D	120.4 ± 28.9	81.9 ± 25.6
**Phenols**	4-vinylguaiacol	1317.45	281.4 ± 11.2	310.9 ± 22.3	238.7 ± 39.4	250.1 ± 167.4	258.7 ± 53.9	287.5 ± 21.8	292.7 ± 17.6	98.8 ± 11.4	118.1 ± 15	284.1 ± 14.5

Compounds detected in *S. eubayanus* fermentation by headspace solid-phase microextraction followed by gas chromatography-mass spectrometry (HS-SPME-GC-MS). SEM: standard error of the mean; CAS: Chemical Abstracts Service; N/D: Not Detected; RI: Kovats retention indices.

**Table 2:** Main Volatile Compounds detected in CL905.1 and CL602.1.

			Relative Abundance (×10^6^) and SEM			
			Strain			
			CL905.1		CL602.1	
Chemical Group	Compound	RI	12 °C	20 °C	12 °C	20 °C
**Aliphatic esters**	Ethyl octanoate	1192.63881	483.1 ± 250.2	1566.67 ± 251.66	228.8 ± 76	1566.67 ± 120.19
	Ethyl 9-decenoate	1384.56268	448.5 ± 165.1	622.93 ± 232.23	137.5 ± 63.5	622.93 ± 116.55
	Ethyl decanoate	1391.77014	1700 ± 734.6	3666.67 ± 1011.60	141.7 ± 38.3	3666.67 ± 409.61
	Isoamyl decanoate	1644.71354	102.1 ± 28.9	162.40 ± 44.51	N/D	162.40 ± 21.72
	Ethyl tetradecanoate	1786.82537	228.2 ± 38.9	267.33 ± 52.60	49.7 ± 6.8	267.33 ± 23.47
	Ethyl hexadecanoate	1991.92208	666 ± 179.7	553.43 ± 59.25	N/D	553.43 ± 48.51
**Aromatic esters**	Phenethyl acetate	1250.12471	522.4 ± 233.1	687.80 ± 328.51	N/D	687.80 ± 189.00
**Aromatic alcohols**	Phenethyl Alcohol	1116.18753	3800 ± 971.5	8600.00 ± 346.41	2900 ± 805	8600.00 ± 851.14
**Aromatic aldehydes**	Benzaldehyde, 4-methyl	1083.5138	N/D	170.50 ± 58.41	42.1 ± 6	170.50 ± 21.10
**Carboxylic acids**	Octanoic acid	1171.38687	633.5 ± 101.3	N/D	12.5 ± 2.4	131.80 ± 13.44
	Dodecanoic acid	1556.73878	38.3 ± 3.4	1866.67 ± 404.15	N/D	1866.67 ± 135.36
**Phenols**	4-vinylguaiacol	1317.44761	238.7 ± 39.4	308.03 ± 76.01	98.8 ± 11.4	290.93 ± 38.64

Compounds detected in *S. eubayanus* fermentation by HS-SPME-GC-MS. SEM: standard error of the mean; CAS: Chemical Abstracts Service; N/D: Not detected; RI: Kovats retention indices.

## Supplementary Materials

The following are available online at https://www.mdpi.com/2076-2607/8/5/755/s1. Figure S1 Fermentation profiles of *S. eubayanus* strains from central and southern Chile in beer wort. The fermentative kinetics of geographically-grouped wild yeast strains represent their fermentative capacity, as measured by CO_2_ loss (g/L). (a) Central strains: Region of Maule, Bío-Bío and La Araucanía. (b) Southern strains: Region of Los Ríos, Los Lagos, and Aysén. (c) Far southern strains: Region of Magallanes. As a negative control, we used beer wort without yeast cells. The commercial lager strain *S. pastorianus* W34/70 was used as a fermentation control. Figure S2. Pearson Correlation between the main volatile compounds identified and latitude. Each point depicts an average of the relative amount produced. Figure S3. Fermentation profiles of CL905.1 and CL602.1 strains in beer wort. The fermentative kinetics of both wild yeast strains represent their fermentative capacity, as measured by CO_2_ loss (g/L). (a) Fermentation at 12 °C. (b) Fermentation at 20 °C. Table S1. *Saccharomyces eubayanus* isolates used in this work. Table S2. Presence/Absence table of Volatile compounds detected in *S. eubayanus*. Table S3. Presence/Absence of Volatile compounds detected in CL905.1 and CL602.1 strain at two fermentation temperatures. Table S4. Total YAN consumption. Table S5. YAN consumption at 24 h of Fermentation. Table S6. YAN consumption at 48 h of Fermentation. Table S7 YAN consumption at 168 h of Fermentation.
