# Supplementary material for: Volatile Compound Screening Using HS-SPME-GC/MS on Saccharomyces eubayanus Strains under Low-Temperature Pilsner Wort Fermentation
Source: Microorganisms. 2020 May 18;8(5):755. doi: 10.3390/microorganisms8050755 (PMC7285299; doi:10.3390/microorganisms8050755)
Supplement: Supplementary file 1 [file microorganisms-08-00755-s001.zip › Supplementary Figures.pdf]

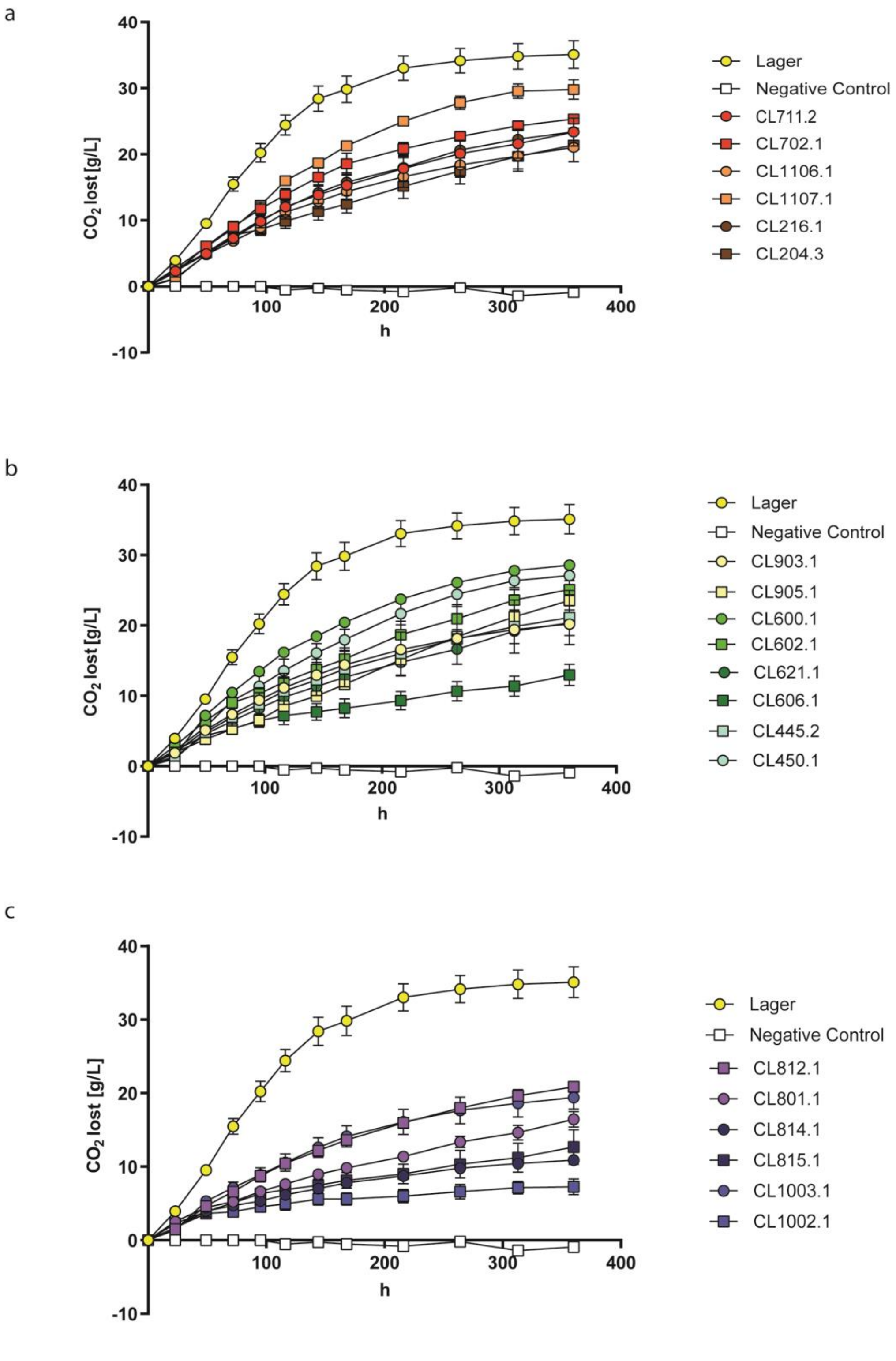

Supplementary Figure 1. Fermentation profiles of *S. eubayanus* strains from central and southern Chile in beer wort. The fermentative kinetics of geographically-grouped wild yeast strains represent their fermentative capacity, as measured by CO_2 loss (g/L). (a) Central strains: Region of Maule, Bío-Bío and La Araucanía. (b) Southern strains: Region of Los Ríos, Los Lagos and Aysén. (c) Far southern strains: Region of Magallanes. As a negative control, we used beer wort without cells. The commercial lager strain *S. pastorianus* W34/70 was used as a fermentation control.

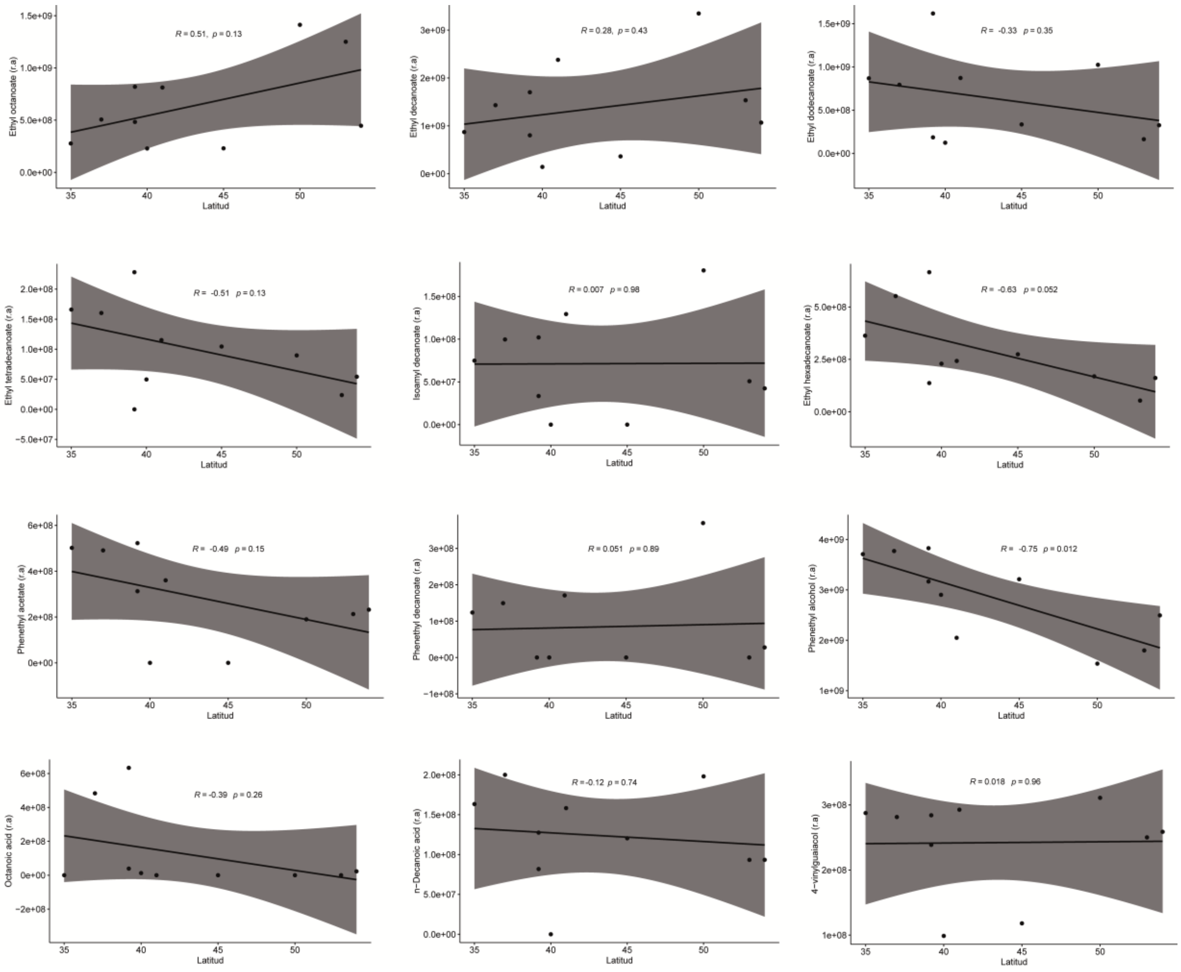

Supplementary Figure 2. Pearson Correlation between the main volatile compounds identified and latitude. Each point depicts an average of the relative amount produced.

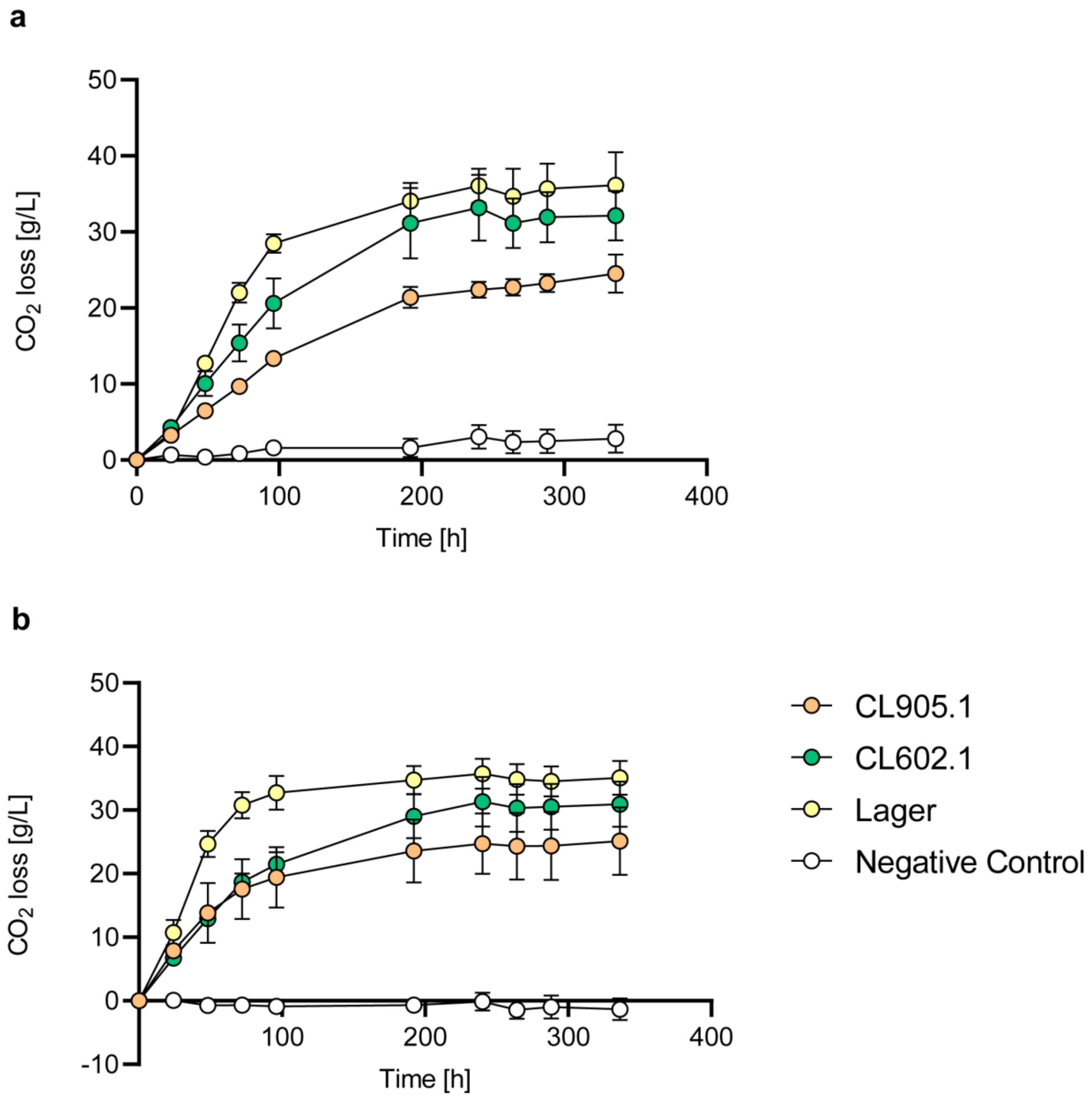

Supplementary Figure 3. Fermentation profiles of CL905.1 and CL602.1 strains in beer wort. The fermentative kinetics of both wild yeast strains represent their fermentative capacity, as measured by CO_2 loss (g/L). (a) Fermentation at 12°C. (b) Fermentation at 20°C.
